# Rupture of Ectopic Ovarian Pregnancy Accompanied by Massive Intra-abdominal Bleeding and Disorder of the Coagulation Mechanism: A Rare and Life-Threatening Obstetric Complication

**DOI:** 10.7759/cureus.28112

**Published:** 2022-08-17

**Authors:** Efthymia Thanasa, Anna Thanasa, Ektoras-Evangelos Gerokostas, Evangelos Kamaretsos, Nikoleta Koutalia, Gerasimos Kontogeorgis, Ioannis Thanasas

**Affiliations:** 1 Medicine, Aristotle University of Thessaloniki, Thessaloniki, GRC; 2 Obsretrics and Gynecology, General Hospital of Trikala, Trikala, GRC; 3 Obstetrics and Gynecology, General Hospital of Trikala, Trikala, GRC

**Keywords:** case report, treatment, diagnosis, hemoperitoneum, rupture, ovarian pregnancy

## Abstract

The rupture of ectopic ovarian pregnancy accompanied by massive intra-abdominal bleeding is a rare obstetric complication, occurs predominantly in the first trimester of pregnancy, and can be dangerous and life-threatening for the pregnant woman. Our case describes a 37yr old woman with a history of 4 lower segment Cesarian sections (LSCS) (Caesarean sections) and multiple surgical abortions, presenting at the ER with acute abdomen symptoms. The patient's hemodynamic status was unstable. The positive urine pregnancy test combined with the clinical and ultrasound findings established the diagnosis of the ruptured ectopic pregnancy, and immediate surgical treatment was decided. During surgery, a large amount of blood was found in the peritoneal cavity, resulting from a rupture of the right ovary and accompanied by hemorrhagic infiltration of adjacent tissues, without participation in the damage of the ipsilateral fallopian tube. It was deemed necessary to remove the ipsilateral adnexa and whole blood transfusion. The patient was discharged from our department on the fourth postoperative day. The price of beta-chorionic gonadotropic hormone was on a downward trend. Three weeks later, the level of beta-chorionic gonadotropic hormone was zero. In the present paper, a brief review is attempted regarding the diagnostic and therapeutic approach for patients with ruptured ectopic ovarian pregnancy after describing the case.

## Introduction

Ectopic pregnancy is common. It is estimated that it concerns about 2% of all pregnancies [1], with the most common type that appears in the fallopian tube (tubal pregnancy) [2]. Today, despite early diagnosis and treatment, which has significantly reduced the morbidity and mortality of the disease, ectopic pregnancy is still responsible for 10% of deaths associated with the first trimester of pregnancy [3,4]. The extra-tubal localization of the disease (ovary, cervix, peritoneal cavity, cesarean section scar) is rare and is associated with about 5% of all ectopic pregnancies [5]. Ovarian pregnancy is the most common type of extra-tubal ectopic pregnancy. In ovarian pregnancy, the implantation of the fertilized ovum may concern the inside of the cortex of the ovary (primary) or the surface of the ovary (secondary ovarian pregnancy). Ovarian pregnancy was first described by Saint Maurice in 1682 [6] and is estimated to account for approximately 3% of all ectopic pregnancies [7].

The present case report highlights the significant difficulties in preoperative diagnosis of ruptured ectopic ovarian pregnancy. Simultaneously, it is pointed out that, despite its rarity, the rupture of ovarian pregnancy must be included among other pathological conditions in the differential diagnosis of women of reproductive age who are presented to the emergency department with acute abdominal pain and symptoms of severe anemia, since early diagnosis and treatment can significantly reduce morbidity and mortality rates.

## Case presentation

The case report concerns a 37-year-old woman with four cesarean sections in her medical history and a history of multiple surgical abortions. The patient, unaware she was pregnant, arrived at the emergency department after a fainting episode. She reported acute stabbing pain in the lower abdomen, sweating, dizziness, and vomiting. Blood pressure was 90/60mmHg and heart rate 110/min. Secondary amenorrhea was evaluated at nine weeks and six days, and the urine pregnancy test was positive. Urgent laboratory examination revealed signs of hemodynamic instability with negative markers of inflammation and mild blood coagulation disorder (Table 1).

**Table 1:** Urgent laboratory examination of ectopic ovarian pregnancy (our case) Signs of hemodynamic instability with negative inflammatory markers and mild blood coagulation disorder are evident (Ht – Hematocrit, Hb – Hemoglobin, PLT – Platelets, WBC – White Blood Cells, NEUT – Neutral, CRP – C Reactive Protein, APTT – Activated Partial Thromboplastin Time, INR – International Normalized Ratio, FIB – Fibrinogen)

Laboratory Tests	Found Laboratory Values	Normal Laboratory Values
Ht	28.9%	37.7 – 49.7%
Hb	9.1 gr/dl	11.8 – 17.8 gr/dl
PLT	227x10^3^/ml	150 – 350 x10^3^/ml
WBC	11.2x10^3^/ml	4 – 10.8 x10^3^/ml
NEUT	77.3%	40 – 75%
CRP	0.51 mg/dl	<0.7 mg/dl
APTT	39.2 sec	24.0 – 35.0 sec
INR	1.29	0.8 – 1.2
FIB	185 mg/dl	200 – 400 mg/dl

**Figure 1:**
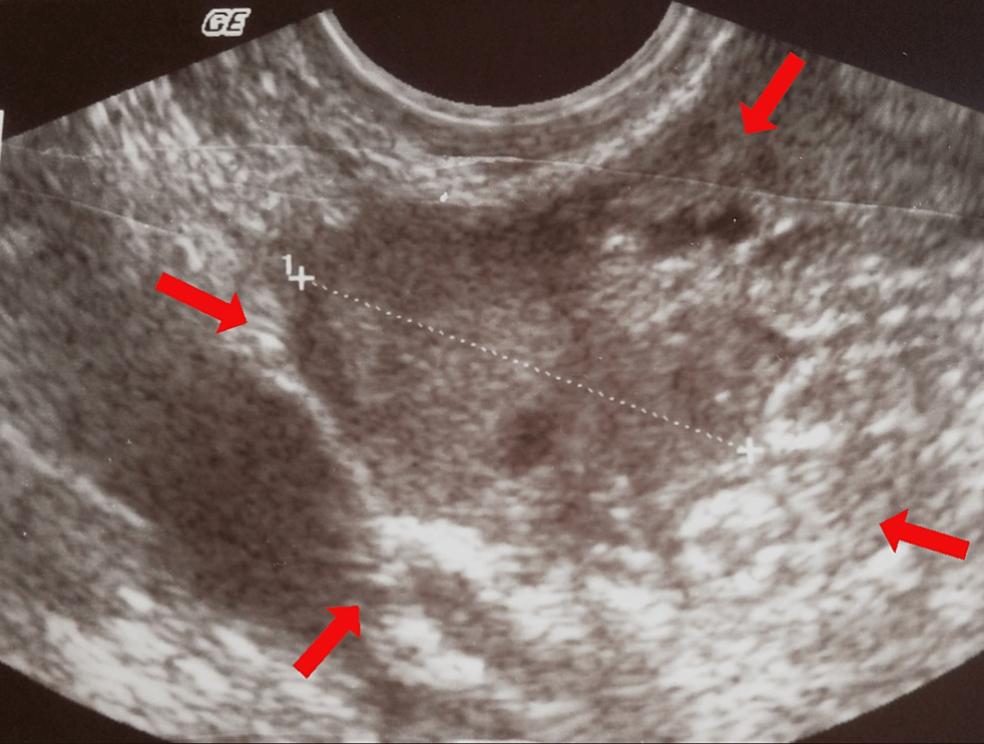
Transvaginal ultrasound imaging of an ectopic ovarian pregnancy (our case) The presence of heterogeneity in the anatomical area of the adnexa (red arrows), combined with the absence of an intrauterine gestational sac, supports the diagnosis of ruptured ectopic pregnancy.

**Figure 2:**
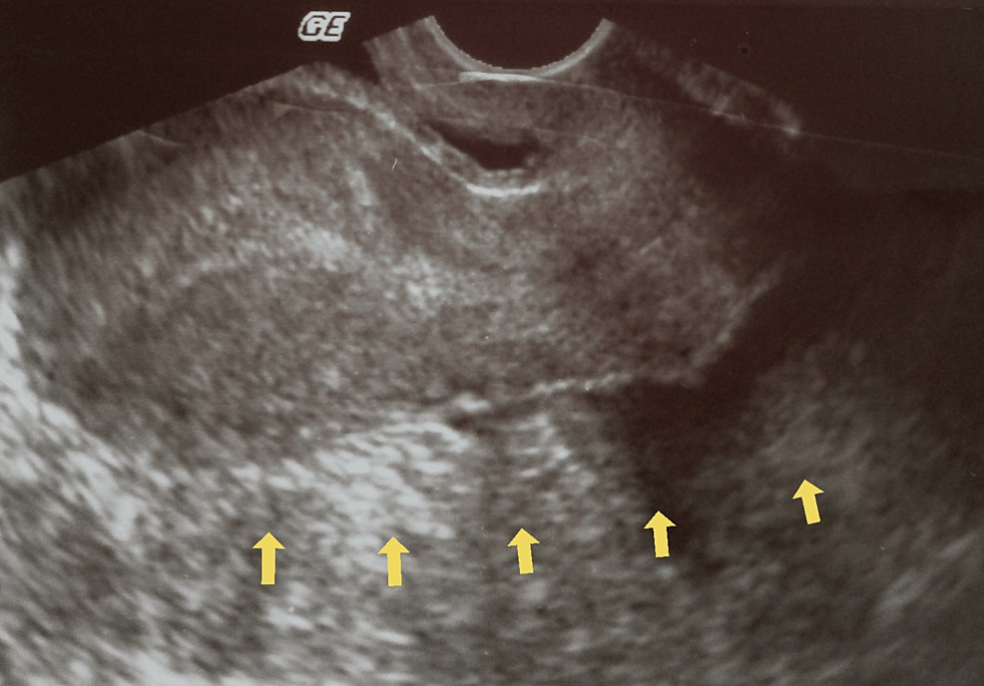
Transvaginal ultrasound imaging of ectopic ovarian pregnancy (our case) The presence of free fluid and blood clots in the cul-de-sac (yellow arrows) and the absence of an intrauterine gestational sac support the diagnosis of ruptured ectopic pregnancy.

Renal function was normal. A quantitative determination of the beta-chorionic gonadotropin hormone was sent. The gynecological examination showed mild vaginal bleeding and great sensitivity to the movement of the cervix. The abdomen was intensely painful on palpation, with signs of peritoneal irritation. Transvaginal ultrasound revealed the presence of inhomogeneity in the anatomical area of the right adnexal (Figure 1), an empty gestational sac in the uterine cavity, free fluid, and multiple blood clots in the pouch of Douglas (Figure 2).

**Figure 3:**
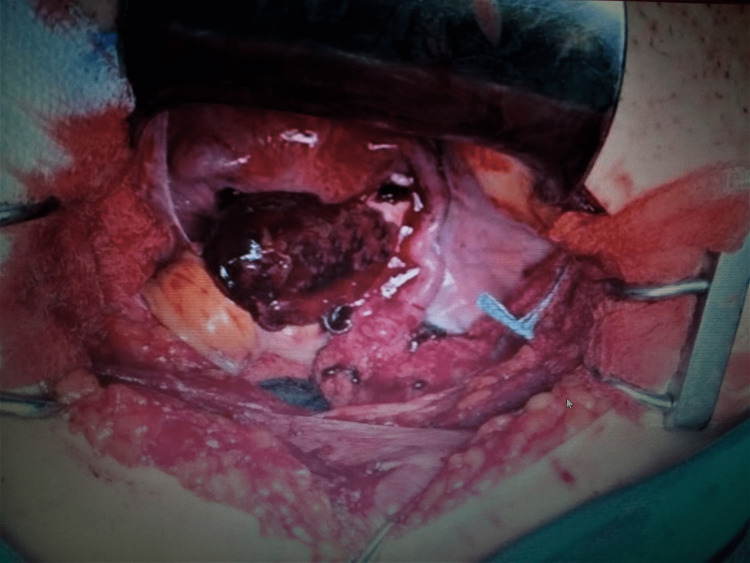
Intraoperative imaging of a ruptured ovarian pregnancy (our case) After cleaning the pelvis from a large amount of blood and blood clots, the ovarian lesion and non-involvement of the corresponding fallopian tube are evident.

Clinical and ultrasound findings and the presence of a positive urine pregnancy test preoperatively established the diagnosis of ruptured ectopic tubal pregnancy. The patient's hemodynamic instability led to the decision for immediate surgical treatment with laparotomy. After opening the abdominal wall, a significant hemoperitoneum was found, and it was deemed necessary to start transfusion with 2 Packed Red Blood Cells and 1 Blood plasma. Intraoperatively, a hemorrhagic rupture of the right ovary was found, without the involvement of the ipsilateral fallopian tube in the damage and the presence of many blood clots in the pelvis (Figure 3). The amount of blood and water used to clean the peritoneal cavity in the suction container was 900ml, while the blood clots removed filled a container with dimensions of 15x10x10 cm.

**Figure 4:**
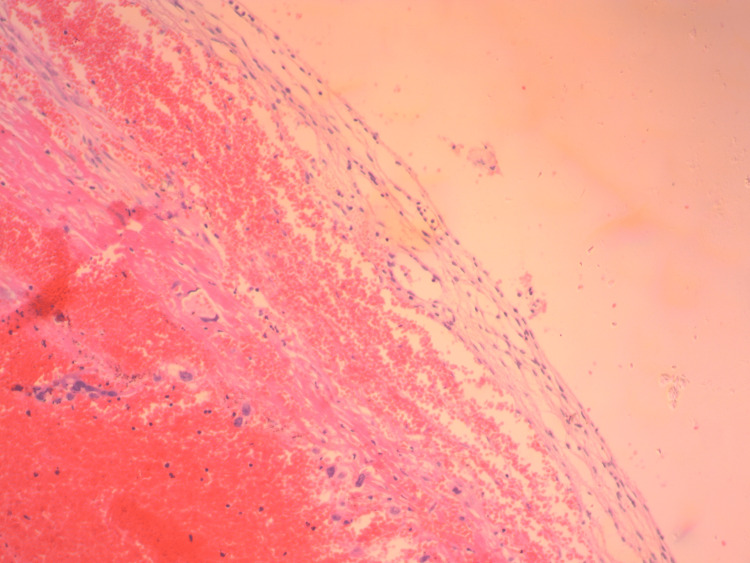
Histological image of a ruptured ectopic ovarian pregnancy (our case) Rare cytotrophoblastic cells are identified within a hemorrhagic substrate

**Figure 5:**
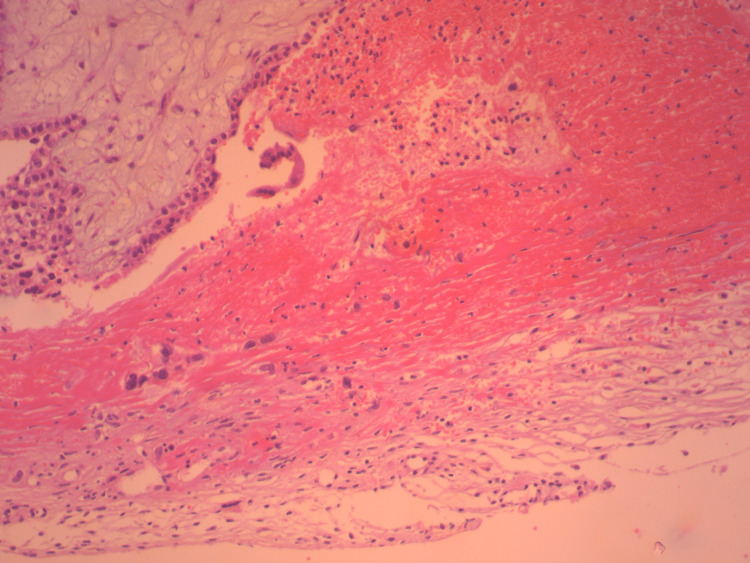
Histological image of a ruptured ectopic ovarian pregnancy (our case) A part of chorionic villi and syncytium formation can be identified within the hemorrhagic substrate

The diagnosis of possible ruptured ectopic ovarian pregnancy was made. The large traumatic surface of the ovary accompanied by hemorrhagic infiltration of the suspensory ligament and the formation of a hematoma at the level of the broad ligament led to the decision to perform salpingo-oophorectomy. Despite the patient's history (the woman was multiparous and had undergone multiple surgical abortions), it was not decided to perform a bilateral salpingectomy because the patient's written and signed consent was not obtained preoperatively. Histological examination of the surgical specimen confirmed the diagnosis (Figures 4, 5).

After a smooth postoperative course, with transfusion of 4 Packed Red Blood Cells and a decreasing value of beta-chorionic gonadotropin hormone, the patient was discharged from our department on the fourth postoperative day. The level of beta-chorionic gonadotropic hormone was on a downward trend; three weeks later, the beta-human chorionic gonadotropin level was zero

## Discussion

The exact etiopathogenic mechanism of the development of ectopic ovarian pregnancy is unknown. The use of contraceptive methods (intrauterine contraceptive devices, hormonal contraception) is estimated to be the leading risk factor for the implantation of the fertilized egg in the ovary. Generally, intrauterine contraceptive devices (19.3%) and fertility treatments (18.1%) remain the most critical risk factors for ectopic ovarian pregnancy [8]. Also, endometriosis, pelvic inflammatory disease, history of ectopic pregnancy, age, and socioeconomic factors are included in the risk factors of ectopic ovarian pregnancy [3,9]. Our patient never received hormonal contraception, nor did she undergo any assisted reproduction treatments. Also, no adhesions in the pelvis were found intraoperatively as a result of the multiple cesarean sections. Rarely, as in our patient, ovarian pregnancy can occur without the presence of classic risk factors [10].

The similar clinical symptomatology of ovarian pregnancy to ectopic tubal pregnancy makes preoperative diagnosis hardly impossible [11]. In our case, the patient was taken to surgery with a preoperative diagnosis, which was incorrectly attributed to a ruptured tubal pregnancy. The deep mild abdominal pain that usually characterizes inextricable ovarian pregnancy, in cases of ovarian rupture and massive intra-abdominal bleeding is replaced by acute abdominal pain accompanied by signs of peritoneal irritation and symptoms of severe anemia (hypotension, pulse acceleration, dizziness, sweating, pallor, fainting episode) [12]. The rupture, which is usually accompanied by massive, life-threatening bleeding, occurs in most cases (91%) during the first trimester. Rupture of ovarian pregnancy in the second and third trimesters is rare and concerns 5.3% and 3.7% of cases, respectively [13]. Also, the surgical management of unruptured ectopic ovarian pregnancy in the second trimester of pregnancy has been described in the literature [14]. The differential diagnosis from the rupture of a fallopian pregnancy, the rupture of a corpus luteum cyst, or the ovarian cyst torsion based on clinical criteria is very difficult [15].

Similarly, the diagnostic value of the imaging test is significantly disputed. Transvaginal ultrasound may have a place in the diagnostic quiver of undisturbed ovarian pregnancy [16]. However, the ultrasound findings are not typical in the case of ruptured ovarian pregnancy and massive bleeding [17]. In some cases, a Computed Tomography scan can help distinguish between a ruptured luteal cyst and ruptured ectopic pregnancy with bleeding [18]. In most cases, the diagnosis of ovarian pregnancy is based on histopathological findings [19]. Even intraoperatively, it is challenging to differentiate ovarian pregnancy from hemorrhagic cyst [20]. Today, based on the modified criteria of Spielberg, the non-involvement of the Fallopian tube and the proven presence of chorionic villi in the ovary confirm the diagnosis of ovarian pregnancy [21].

Treatment of ovarian pregnancy (pharmacological or surgical with laparotomy or laparoscopic access) depends on the time of diagnosis and the patient's hemodynamic status. Treatment with methotrexate is not an option in cases of massive bleeding. Similarly, laparotomy seems to be preferred to the laparoscopic approach by most surgeons, although cases of laparoscopic treatment have been reported in patients with hemoperitoneum [22]. The traditional surgical treatment with laparotomy or laparoscopic access consists of wedge resectioning of the ovary. For those cases where the diagnosis is made late and is accompanied by severe bleeding, it may be necessary to perform an oophorectomy or salpingo-oophorectomy [23]. In our case, salpingo-oophorectomy was deemed necessary after hemorrhagic infiltration and hematoma formation in the corresponding parametrium. There is usually a reasonable prognosis of ovarian pregnancy. No case of recurrent ectopic ovarian pregnancy has been reported in the literature [24]. Spontaneous bleeding after the rupture of ovarian pregnancy is the main complication, with mortality at fairly high rates [25].

## Conclusions

The rupture of ovarian pregnancy accompanied by massive intra-abdominal bleeding is a rare obstetric complication that mainly concerns the first trimester of pregnancy and can be dangerous and life-threatening for the pregnant woman. Preoperative diagnosis is a challenge in contemporary obstetric clinical practice since it is usually misdiagnosed as a ruptured fallopian tube. In any case, the rupture of the ovarian pregnancy should be included in the differential diagnosis of pregnant women who are in the first trimester of pregnancy and presented with acute abdominal pain and symptoms of severe anemia to reduce the increased risk of maternal morbidity and mortality.
